# Family transfers and long-term care: An analysis of the WHO Study on global AGEing and adult health (SAGE)

**DOI:** 10.1016/j.jeoa.2017.08.003

**Published:** 2018-11

**Authors:** Adrianna Murphy, Paul Kowal, Marco Albertini, Bernd Rechel, Somnath Chatterji, Kara Hanson

**Affiliations:** a London School of Hygiene and Tropical Medicine, UK; b World Health Organization, United States; c University of Newcastle, Australia; d University of Bologna, Italy; e European Observatory on Health Systems and Policies, UK

## Abstract

**Background:**

Populations globally are ageing, resulting in increased need for long-term care. Where social welfare systems are insufficient, these costs may fall to other family members. We set out to estimate the association between long-term care needs and family transfers in selected low- and middle- income countries.

**Methods:**

We used data from the World Health Organization’s Study on global AGEing and adult health (SAGE). Using regression, we analysed the relationship between long-term care needs in older households and i) odds of receiving net positive transfers from family outside the household and ii) the amount of transfer received, controlling for relevant socio-demographic characteristics.

**Results:**

The proportion of household members requiring long-term care was significantly associated with receiving net positive transfers in China (OR: 1.76; p = 0.023), Ghana (OR: 2.79; p = 0.073), Russia (OR: 3.50; p < 0.001). There was a statistically significant association with amount of transfer received only in Mexico (B: 541.62; p = 0.010).

**Conclusion:**

In selected LMICs, receiving family transfers is common among older households, and associated with requiring long-term care. Further research is needed to better understand drivers of observed associations and identify ways in which financial protection of older adults’ long-term care needs can be improved.

## Introduction

Lower fertility and mortality rates have resulted in population ageing globally, presenting most countries with the challenge of sustaining economic growth while supporting their older adult populations (Lee et al., 2010). About two-thirds of the global population of adults aged 60 years and older reside in low- and middle-income countries (LMICs). Meeting the needs of ageing populations in these contexts may be particularly difficult when doing so requires not only resources, but also the establishment of social welfare systems that ensure the needs of older adults are met, for example through the provision of long-term care or health insurance and pensions (Lee et al., 2010). In many LMICs, welfare systems have only partially and insufficiently adapted to the needs of an ageing population, although there are exceptions, such as Namibia, which provides a universal, non-means tested pension to people older than 60 years (World Health Organization, 2015). Where public transfer systems are weak, their role is often filled by private intergenerational family financial transfers (from adult children to ageing parents) (Lee et al., 2010). This may be particularly so in LMICs where norms of filial obligation to support parents are stronger than in western high-income countries (Lee, 1994, Rajan and Kumar, 2003, Ching Ying Ng et al., 2002).

A major source of costs among ageing populations is the need for long-term care due to ill health or disability. The health needs of ageing societies are dominated by chronic, non-communicable diseases (NCDs), which often impair ability to perform activities of daily living and tend to require long-term (often life-long) care and medical treatment. Ageing populations are also at higher risk of chronic condition multi-morbidity (suffering from two or more chronic conditions at the same time) (Barnett et al., 2012, Salive, 2013), exposing them to even higher long-term care costs. In LMICs, where health care coverage is typically not universal or comprehensive, costs associated with long-term care for chronic illness or disability incurred by households can lead to catastrophic spending and impoverishment (Xu et al., 2003, Murphy et al., 2013, Goryakin and Suhrcke, 2014). Previous research has suggested that, when faced with costs of care for chronic illnesses, households may cope by accepting financial or in-kind transfers from family outside the household (Murphy et al., 2013, Alam and Mahal, 2014, Kruk et al., 2009, Flores et al., 2008). The extent to which households with long-term care needs rely on family transfers is likely to vary from country to country, depending on the social welfare system and the burden of costs of care borne by families. For example, a 16-country wide study in Europe showed that greater pension entitlement reduced inequality in unmet health care need among older adults, especially in countries with health systems funded largely by out-of-pocket payments (Reeves et al., 2017). Our aim was to test for a relationship between long-term care needs and family transfers in LMICs with different health and social welfare systems.

Family transfers are also likely driven by other socio-demographic factors that are related to health and long-term care needs. Transfers are likely to vary with age (Lee et al., 2010), economic (Dhanaraj, 2016, Leive and Xu, 2008, Nguyen et al., 2012), and education status of the household (Albertini et al., 2007), as well as with unemployment, when labour income is no longer available to meet material needs (Lee et al., 2010). They are also likely affected by gender, for example in cases where widows are unemployed, no longer collecting their husbands' pension (Lee et al., 2010), and more likely to rely on others to finance health care expenditure (Onah and Govender, 2014). Transfers to finance long-term care needs are also likely to vary by health insurance status, although this relationship may depend on the comprehensiveness of insurance benefits, and how much households are left to pay out-of-pocket (Mills, 2014). Other socio-cultural factors have been put forth as possible determinants of family transfers, such as values, traditions and practices (Albertini et al., 2007). While we cannot operationalize these with the data at our disposal, we expect some of these to be captured by the inclusion of six different LMICs, as well as the inclusion of an urban/rural variable in our analysis. Research has shown that norms of filial obligation may be stronger in rural areas (Qi, 2014), and that health care needs and utilization vary by place of residence (Chow et al., 2013).

In the long term, coping with health care costs using family transfers may negatively affect the economic security of the household and their extended families (Flores et al., 2008, Russell, 1996, Wagstaff, 2008). Furthermore, since the economic well-being of different family generations can be highly correlated (Solon, 1992), this form of financing health and long-term care needs will likely exacerbate existing inequalities in quality of life and life expectancy (Ross and Mirowsky, 2002). Understanding patterns of family transfers in LMICs, and their association with the long-term care needs of households may provide insights into the extent to which the financial burden of long-term care needs of ageing populations is borne by families. However, existing research on family transfers is often limited to high-income countries (Albertini et al., 2007, Silverstein and Giarrusso, 2010, Lin and Wu, 2014, Mellor, 2001), or fails to quantify the extent of family transfers and their association with long-term care needs of households (Kruk et al., 2009). We set out to describe patterns of family transfers to older households in six LMICs (China, Ghana, India, Mexico, Russia and South Africa) and to estimate the association between these transfers and the long-term care needs of these households, including in our analysis other potential determinants of family transfers.

## Methods

We used data from the World Health Organization’s Study on global AGEing and adult health (SAGE) Wave 1 (World Health Organization, 2016). The methods used in WHO SAGE have been described previously (Kowal et al., 2012). Briefly, SAGE is a longitudinal study of ageing and health that includes nationally representative samples of individuals aged 50 years and older, and smaller comparison samples of younger adults (aged 18–49). For Wave 1, face-to-face interviews were conducted in China (2008–10), Ghana (2008–09), India (2007–08), Mexico (2009–10), the Russian Federation (2007–10) and South Africa (2007–08). Households were sampled using multi-stage cluster sampling and household enumerations were carried out for the final sampling units. One household questionnaire was completed per household. In households selected to have an individual interview with a member aged 50 and older, all those aged 50 and older were invited to participate. Proxy respondents were identified for selected individuals who were unable to complete the interview for health or other reasons. Household-level analysis weights and person-level analysis weights were generated for each country.

Household wealth quintiles for WHO SAGE were generated using an asset-based approach through a multi-step process. The assets were derived from the household ownership of durable goods, dwelling characteristics (type of floors, walls and cooking stove), and access to services such as improved water, sanitation and cooking fuel. Durable goods included number of chairs, tables or cars, and if, for example, the household had electricity, a television, fixed line or mobile phone, or washing machine. A total of 21 assets were included with overlaps and differences in the asset lists by country. Resulting data were reshaped so that a pure random effect model could be used to generate specific thresholds on the latent income scale, and country-specific “asset ladders”. A Bayesian post-estimation (empirical Bayes) method was used to then convert the asset ladder so that raw continuous income estimates could be transformed into quintiles. The process of deriving country-specific asset ladders and transforming these into wealth quintiles has been explained in detail previously (Kowal et al., 2012). The income quintile variable was generated from the unweighted data – so distributions will shift when accounting for the complex survey design.

For this study, data from the household roster were used to identify households where all members were aged 50 years or older. This resulted in an analytical sample of n = 8700 households (the number of households in each country is shown in Table 1). The mean household size of households aged 50 years or older in each country was: China 1.72 (95% CI: 1.70–1.74); Ghana 1.22 (1.18–1.26); India 1.70 (1.66–1.74); Mexico 1.90 (1.87–1.93); Russia 1.51 (1.49–1.53); South Africa 1.40 (1.36–1.44). Among these households, the proportion of household members requiring long-term care was estimated. A household member was defined as requiring long-term care if the household informant answered positively to the following question for that household member: “Does *[NAME]* need care due to his/her health condition, such as a long-term physical or mental illness or disability, or because he/she is getting old and weak?”. The net mean total monetary value of transfers (financial + in-kind) received by households was estimated in each of the six countries. Financial transfers were defined by self-reported estimates of cash received by the household from family outside the household, net of transfers to family living outside the household, over the 12 months preceding interview. In-kind transfers (into and out of the household) were defined by the self-reported estimate of the value of any non-monetary goods received/provided by the household from/to family outside the household. These were then divided by the number of household members to provide a per capita transfer estimate. Local currency was converted to $USD using World Bank average exchange rates for the year of data collection in each country.

**Table 1:** Characteristics of analytical sample (mean or percent with 95% confidence intervals) for older adult households, by country and income quintiles, WHO Study on global AGEing and adult health (SAGE) Wave 1 (2007/10).

Households aged 50+ (n = number of households)	Income quintile distribution (%)*	Mean household age	Mean proportion of household needing care (%)	Households with net positive transfers (%)	Households with net negative transfers (%)	Mean net per capita family transfer (past 12 months, USD, only those who received or gave)
China (n = 4429)						
Poorest	24.0 (21.1, 27.1)	60.2 (67.3, 69.1)	4.5 (2.9–6.1)	55.9 (52.1,59.6)	3.2 (2.1,5.0)	144.85 (125.81,163.89)
2	19.2 (17.0, 21.7)	65.9 (65.1, 66.6)	3.5 (2.5–4.4)	51.6 (46.9,56.3)	6.0 (4.4,8.0)	152.42 (87.54, 217.31)
3	21.3 (19.4,23.3)	64.6 (63.4, 65.8)	2.7 (2.0–3.7)	38.0 (33.4, 42.8)	14.0 (11.7,16.6)	42.83 (−38.63,124.30)
4	21.6 (18.5, 25.0)	63.4 (62.1, 64.7)	2.3 (1.3–3.3)	35.6 (29.1, 42.6)	16.9 (13.9, 20.4)	−15.67 (−97.66, 66.32)
Richest	13.9 (11.3, 16.9)	63.7 (62.0, 65.5)	2.6 (1.1–4.1)	16.0 (11.6, 21.8)	16.6 (10.9, 24.5)	−244.15 (−588.23, 99.93)

Ghana (n = 575)						
Poorest	30.3 (26.0,34.7)	67.6 (66.0, 69.2)	6.2 (2.8, 9.6)	55.2 (47.9, 62.5)	5.0 (1.0, 9.0)	38.06 (22.28, 53.83)
2	24.9 (21.1, 28.7)	69.3 (67.3, 71.4)	3.9 (0.7, 7.1)	63.9 (55.7, 72.1)	9.8 (4.9, 14.7)	36.48 (10.13, 62.83)
3	21.7 (18.2, 25.2)	63.3 (61.3, 65.2)	4.1 (0.6, 7.6)	42.4 (32.2, 52.6)	20.0 (10.8, 29.1)	20.26 (2.34,38.17)
4	15.3 (11.6, 18.9)	64.2 (62.0, 66.4)	2.2 (0.0, 5.5)	58.0 (46.5, 69.6)	17.6 (8.9, 26.3)	45.27 (13.77, 76.78)
Richest	7.8 (5.3,10.2)	63.6 (60.8, 66.3)	6.6 (0.0, 13.1)	52.9 (36.0, 69.7)	36.6 (19.7, 53.5)	−38.09 (−145.18, 69.00)

India (n = 391)						
Poorest	51.6 (43.1, 60)	66.1 (64.6, 67.6)	3.3 (1.2, 5.5)	40.6 (31.5, 49.8)	3.8 (0.6, 7.1)	54.86 (25.35, 84.37)
2	20.5 (15.3, 25.8)	63.7 (62.1, 65.3)	5.8 (1.5, 10.2)	37.4 (26.2, 48.7)	14.1 (4.9, 23.4)	86.78 (34.38, 139.18)
3	12.6 (7.5, 17.8)	63.8 (61.3, 66.2)	2.5 (−0.4, 5.5)	26.1 (10.7, 41.5)	11.8 (1.7, 22)	62.23 (−9.53, 134)
4	6.1 (3.7, 8.4)	64.8 (62.5, 67)	9.5 (0.9, 18.1)	30.3 (13.2, 47.5)	22.6 (7.5, 37.7)	51.02 (−50.65, 152.68)
Richest	9.2 (4.4, 14)	63.5 (59.9, 67.1)	0.7 (−0.8, 2.3)	19.3 (3.4, 35.2)	43.1 (16.8, 69.4)	153.72 (−309.51, 616.96)

Mexico (n = 577)						
Poorest	31.3 (25.7, 37.0)	72.6 (70.9, 74.2)	9.3 (3.7, 15.0)	36.9 (26, 47.9)	3.2 (−0.3, 6.8)	360.63 (144.96,576.30)
2	25.0 (20.1, 29.9)	72.9 (71.1, 74.7)	13.9 (7.5, 20.2)	46.5 (35.7, 57.3)	4.4 (0.5, 8.2)	437.94 (272.25, 603.64)
3	18.3 (13.3, 23.3)	71.5 (69.4, 73.5)	18.8 (7.9, 29.7)	23.7 (12.8, 34.7)	3.4 (−0.5, 7.3)	251.28 (80.27, 422.29)
4	16.3 (12.2, 20.3)	68.2 (65.6, 70.6)	16.3 (7.7, 24.8)	35.8 (20.9, 50.7)	8.5 (−1.3, 18.2)	239.53 (42.22, 436.85)
Richest	9.0 (5.6, 12.5)	69.1 (66.8, 71.5)	6.2 (0.6, 11.7)	52.1 (32.1, 72.1)	9.1 (0.2, 17.9)	305.94 (−122.58, 734.47)

Russia (n = 1864)						
Poorest	25.3 (18.9, 31.7)	71.6 (68.5, 74.6)	22 (15.5, 28.5)	19.1 (11.7, 26.6)	8.1 (4.3, 11.8)	−116.29 (−506.29, 273.71)
2	24.8 (18.3, 31.4)	70.1 (68.5, 71.7)	25.8 (15.7, 35.9)	26.2 (15.7, 36.8)	10.9 (2.6, 19.2)	−72.73 (−153.75, 8.29)
3	22.4 (17.5, 27.2)	69.9 (68, 71.9)	13 (4.7, 21.2)	24 (14.9, 33.1)	10.4 (6.3, 14.5)	−20.22 (−231.64, 191.21)
4	15.0 (10.5, 19.6)	64.2 (61.2, 67.2)	13 (5.3, 20.7)	20.9 (12.6, 29.2)	24.3 (8.3, 40.4)	−155.27 (−299.09, −11.45)
Richest	12.4 (8.3, 16.5)	63.3 (61, 65.5)	7.3 (1.1, 13.5)	16.5 (6.1, 27)	38.3 (17.8, 58.9)	−863.65 (−1634.65, −92.65)

South Africa (n = 864)						
Poorest	27.3 (20.7, 33.9)	61.7 (58.4, 65.1)	0.7 (−0.6, 2)	2.4 (0.6, 4.3)	8.7 (3.8, 13.6)	−1392.47 (−2944, 159.05)
2	19.7 (15.4, 24)	58.3 (57, 59.7)	0.7 (−0.2, 1.7)	6.6 (1.5, 11.7)	25.2 (16.7, 33.7)	−1361.85 (−1764.99, −958.7)
3	21 (15.4, 26.6)	62 (58.5, 65.5)	1.1 (−0.1, 2.4)	4.5 (1.7, 7.3)	20.2 (11, 29.4)	−1576.47 (−2265.9, −887.05)
4	16.3 (10.9, 21.6)	62.9 (60.1, 65.6)	0.9 (−0.3, 2)	7.9 (1.9, 13.9)	12.4 (1.8, 23.1)	−3.72 (−138.62, 131.19)
Richest	15.7 (12.1, 19.3)	63.6 (61.7, 65.4)	0.3 (−0.1, 0.7)	8.2 (1.1, 15.3)	9.5 (3.9, 15.1)	−1740.7 (−2965.85, −515.56)

Weighted logistic regression models were used to estimate the association between a net positive (1) or net negative or zero (0) per capita family transfer and the proportion of household members requiring long-term care. We used *net* transfers rather than transfers *in* to account for the sometimes large financial or in-kind flows *out* of older households to their adult children, which also vary by household characteristics (Lee et al., 2010). As discussed above, family transfers may be influenced by household characteristics such as economic status and age. To account for the possible confounding effect of these characteristics on the relationship between long-term care needs and family transfers, the model controlled for: i) wealth quintile; ii) urban or rural place of residence; iii) mean household age; iv) proportion of the household currently working; v) highest level of education attained in the household; vi) proportion of the household that is male; and, vii) proportion of the household not covered by health insurance. Finally, weighted linear regression models were used to estimate the association between the financial value of per capita family transfer received, among those households that received net positive transfers, and the proportion of household members requiring long-term care, controlling for the same variables as the above model. All regression models were run separately for each country.

## Results

The income quintile distribution, as well as the mean age, proportion needing long-term care, proportion with net positive and net negative family transfers, and mean net per capita family transfer received are shown in Table 1. In China, the proportion of household members requiring long-term care was highest among the poorest income quintile (4.5%, 95% CI: 2.9%, 6.1%), as was the proportion of households with net positive transfers (55.9%, 95% CI: 52.1%, 59.6%). In Russia as well, the proportion of the household needing long-term care was higher in the poorest two income quintiles (22%, 95% CI: 15.5%, 28.5% and 25.8%, 95% CI: 15.7%, 35.9%) than in the richest (7.3%, 95% CI 1.1%, 13.5%), but there was no pattern between income quintile and proportion receiving net positive transfers. In India, the proportion of households receiving net positive transfers appeared to decrease with income, with 40.6% (95% CI: 31.5%, 49.8%) of the poorest income quintile receiving net positive transfers, compared to only 19.3% (95% CI: 3.4%, 35.2%) in the richest quintile. In all the countries included, except for South Africa, the proportion of households with net negative transfers was highest in the richest or second richest income quintile.

**Fig. 1:**
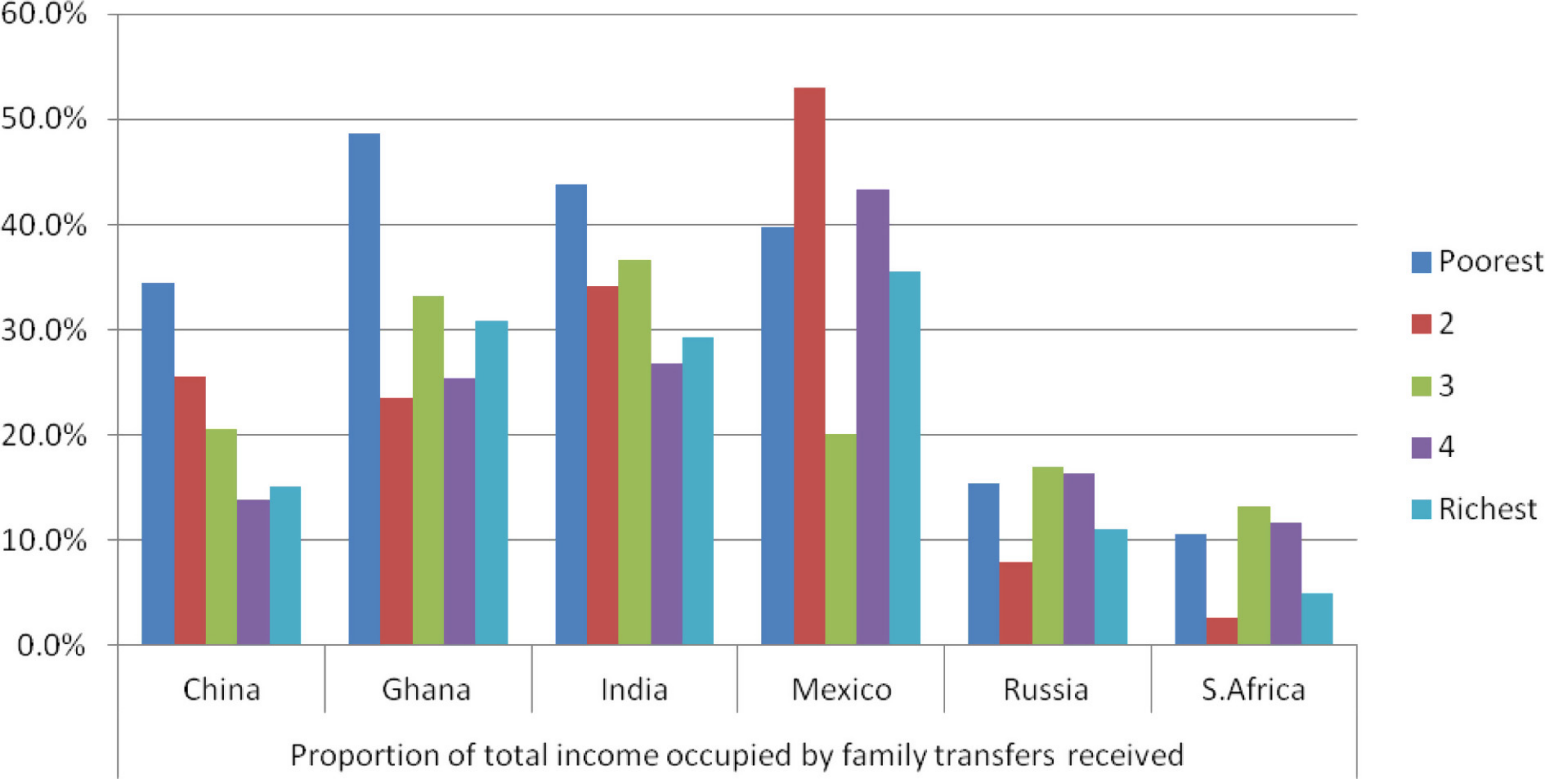
Percentage of household income occupied by family transfers, households 50+ years for each country, by income quintile, WHO SAGE Wave 1 (2007/10).

The per capita value of transfers received (among households with net positive transfers), as a share of total annual per capita household income is shown in Fig. 1. Overall, this share appeared smallest in Russia and South Africa, relative to other included countries. In China, Ghana and India, the transfers occupied the largest share of income in the poorest income quintiles.

Table 2 shows the results of the logistic regression, estimating the association between the proportion of household members requiring long-term care and net negative (0) or net positive (1) transfers, adjusted for covariates. Odds ratios (OR) above 1.00 indicate an increase in odds of net positive transfers vs. net negative transfers associated with a one-unit increase in that variable. As shown in Table 1, the proportion of household members requiring long-term care was low in South Africa, with many households reporting 0%. As a result, there was very little variation in the explanatory variable (proportion needing care). Regression analyses using this variable generated unstable estimates, and therefore results for South Africa are not included here. (The reason for low numbers of older South Africans reporting requiring long-term care requires further investigation, but may be due to how long-term care needs are defined in this population. For example, many older South Africans may receive informal care from extended families (Schatz et al., 2015), which they may not define as long-term care, as opposed to formal care in health facilities, which has been historically less accessible to the majority of the population (Kon and Lackan, 2008). After controlling for income quintile, place of residence, mean household age, proportion male, proportion working, proportion with no health insurance, and highest level of education attained, the proportion of household members requiring long-term care in each country was associated with increased odds of receiving net positive transfers. This result was statistically significant in China (OR: 1.76; p = 0.023), Ghana (OR: 2.79; p = 0.073), and Russia (OR: 3.50; p < 0.001). Odds of net positive transfers were also statistically significantly associated with some income quintiles and place of residence in China and Russia, with mean household age in China, Ghana, India and Mexico, with proportion male in China and Ghana, with proportion working in China, Ghana, India, and Russia, and with highest education level in China

**Table 2:** Association between long-term care needs and net positive per capita family transfers vs net negative or zero per capita family transfers among households 50 years and older, controlling for covariates, by country and income quintiles, WHO SAGE Wave 1 (2007/10).

	Association with net positive vs. net negative or zero per capita family transfer				
	China	Ghana	India	Mexico	Russia
	OR (p-value)	OR (p-value)	OR (p-value)	OR (p-value)	OR (p-value)
**Proportion of household needing long-term care**	1.76	2.79	1.26	1.73	3.50
	(0.023)	(0.073)	(0.733)	(0.203)	(<0.001)
**Income quintile**					
*Poorest*	ref				
*2*	1.29	1.50	0.96	1.27	1.37
	(0.024)	(0.117)	(0.908)	(0.468)	(0.185)
*3*	1.14	0.78	0.81	0.49	1.66
	(0.286)	(0.395)	(0.699)	(0.084)	(0.047)
*4*	1.34	1.45	0.95	1.00	1.63
	(0.026)	(0.300)	(0.922)	(0.995)	(0.140)
*Richest*	0.92	0.98	0.56	2.45	1.44
	(0.573)	(0.957)	(0.434)	(0.071)	(0.375)
**Urban (vs rural)**	0.13	0.74	0.87	0.83	2.96
	(<0.001)	(0.168)	(0.765)	(0.501)	(<0.001)
**Mean household age**	1.04	1.06	1.04	1.04	0.99
	(<0.001)	(<0.001)	(0.033)	(0.011)	(0.286)
**Proportion males**	0.46	0.29	0.62	1.14	0.60
	(<0.001)	(<0.001)	(0.334)	(0.778)	(0.143)
**Proportion working**	0.79	0.48	0.29	1.25	0.47
	(0.042)	(0.002)	(0.003)	(0.653)	(0.006)
**Proportion with no health insurance**	1.28	1.03	3.79	0.95	0.24
	(0.097)	(0.902)	(0.089)	(0.878)	(0.303)
**Highest level of education attained in household**					
*No formal education*	ref				
*Less than primary*	0.86	1.09	1.18	omitted*	0.09
	(0.232)	(0.767)	(0.685)		(0.061)
*Primary completed*	0.61	1.27	0.48	1.01	0.70
	(<0.001)	(0.501)	(0.121)	(0.972)	(0.750)
*Secondary completed*	0.59	1.05	0.97	1.15	0.46
	(<0.001)	(0.924)	(0.949)	(0.803)	(0.484)
*High school completed*	0.44	1.28	0.79	0.53	0.39
	(<0.001)	(0.421)	(0.722)	(0.303)	(0.396)
*Tertiary completed*	0.37	2.30	1.02	0.89	0.47
	(<0.001)	(0.141)	(0.981)	(0.845)	(0.495)
*Post graduate completed*	omitted*	omitted*	no observations	0.76	0.73
				(0.643)	(0.819)
Constant	0.29	0.06 (0.003)	0.02	0.03	0.50
	(0.002)		(0.011)	(0.006)	(0.624)
Observations**	4399	572	378	575	1862

* This level of education perfectly predicted failure (i.e. all households at this level did not receive net positive transfers).

** This number varies slightly from that in Table 1, as any households who lacked complete data on variables in the regression were excluded.

The results of our linear regression to estimate the association between the proportion of household members needing long-term care and amount of family transfer received, among households with net positive transfers and adjusting for socio-demographic variables, are shown in Table 3. Once conditioned on having received net positive transfers, the proportion of household members requiring long-term care did not have a statistically significant effect on amount of transfer received in any country, except for Mexico (B: 541.62; p = 0.010). Statistically significant effects were observed for some income quintiles in China and Ghana, for mean household age in India (negative relationship), proportion male in China (negative relationship), proportion working in Ghana (negative relationship) and some levels of education in Ghana and India

**Table 3:** Estimated association between net amount of per capita family transfer received and proportion of household needing long-term care, among households 50 years and older reporting to have received family transfers, by country and income quintiles, WHO SAGE Wave 1 (2007/10).

	Association with amount of per capita family transfer received				
	China	Ghana	India	Mexico	Russia
	B (p-value)	B (p-value)	B (p-value)	B (p-value)	B (p-value)
**Proportion of household needing long-term care**	4.50	−2.99	−91.12	541.62	119.90
	(0.960)	(0.914)	(0.160)	(0.010)	(0.290)
**Income quintile**					
*Poorest*	ref	ref	ref	ref	ref
*2*	52.86	14.93	65.95	170.93	−181.99
	(0.030)	(0.300)	(0.170)	(0.280)	(0.050)
*3*	85.30	16.00	94.84	−29.93	−63.56
	(0.080)	(0.283)	(0.100)	(0.840)	(0.610)
*4*	41.99	45.61	95.09	140.95	−215.14
	(0.320)	(0.042)	(0.205)	(0.410)	(0.090)
*Richest*	315.73	53.85	409.62	149.61	−175.44
	(<0.001)	(0.120)	(0.250)	(0.400)	(0.170)
**Urban (vs rural)**	48.56	13.92	53.22	85.86	−207.16
	(0.240)	(0.252)	(0.400)	(0.450)	(0.150)
**Mean household age**	−62.26	−1.01	−124.13	−91.02	128.78
	(0.060)	(0.123)	(0.020)	(0.460)	(0.280)
**Proportion males**	−7.13	22.62	1.04	8.42	−3.41
	(<0.001)	(0.165)	(0.810)	(0.430)	(0.560)
**Proportion working**	−8.52	−46.70	−46.66	47.33	−228.06
	(0.810)	(<0.001)	(0.720)	(0.720)	(0.140)
**Proportion with no health insurance**	−32.09	8.92	−109.70	6.74	−78.37
	(0.450)	(0.457)	(0.250)	(0.970)	(0.600)
**Highest level of education attained in household**					
*No formal education*	ref	ref	ref	ref	ref
*Less than primary*	−32.95	−28.81	50.30	omitted*	omitted*
	(0.250)	(0.056)	(0.410)		
*Primary completed*	−57.05	−19.10	34.17	3.75	65.89
	(0.060)	(0.430)	(0.570)	(0.970)	(0.500)
*Secondary completed*	−8.39	−12.26	−65.74	−130.11	117.98
	(0.870)	(0.752)	(0.370)	(0.630)	(0.240)
*High school completed*	17.89	−38.69	−4.12	122.41	222.06
	(0.850)	(0.038)	(0.960)	(0.680)	(0.060)
*Tertiary completed*	437.69	34.69	1100.34	−183.16	185.00
	(0.200)	(0.571)	(0.020)	(0.470)	(0.090)
*Post graduate completed*	no observations	omitted*	no observations	−137.72	169.67
				(0.54)	
Constant	719.24	132.77	105.32	−428.14	553.37 (0.230)
	(<0.001)	(0.009)	(0.720)	(0.550)	
Observations	1476	280	139	147	281

* This level of education perfectly predicted failure (i.e., all households at this level did not receive net positive transfers).

## Discussion

As societies in LMICs become older, research is required to understand how costs for increased long-term care needs are being met, and specifically what burden of long-term care expenditure rests on families. To the best of our knowledge, this is the first study that investigates family transfers to older households across several LMICs and estimates the relationship between receiving transfers and requiring long-term care.

Our findings suggest that a high proportion of households aged 50 years or more in China, Ghana, India, Mexico and Russia, received net positive financial or in-kind transfers from family outside the household. In South Africa, the proportion of households with net negative transfers was higher, which may be in part due to social protection structures in this country. These include a means-tested, non-contributory pension scheme providing income for 75% of the older adult population in retirement, and higher access to basic health care for older adults than in the other five SAGE countries) (Goeppel et al., 2016). There is also evidence that pensions of older South Africans can be an important source of income for some extended families, (Schatz et al., 2015, Lloyd-Sherlock and Agrawal, 2014); when given to women, there is evidence that these pensions have a positive impact on the health of their grandchildren (Duflo, 2000). However, further research is required to understand the higher proportion of households with net negative transfers in this country. The share of household income occupied by net positive transfers was highest in the poorest households in China, Ghana and India. While social security systems in these three countries continue to evolve, problems with inequitable coverage and benefits remain (Fang et al., 2016, Kpessa, 2011, Virk and Atun, 2015). In China in particular, in 2008, the national pension scheme covered only 7.8% of the rural population and while those below the poverty line were eligible for public transfers, these were limited in amount (Wu and Li, 2014).

Our regression results point to a relationship between receiving net positive transfers and requiring long-term care in all included countries, with a statistically significant relationship in China, Ghana, Russia. However, the proportion of those insured in the household was not associated with receiving net positive transfers. Together, these results suggest that in these countries, requiring long-term care leads to increased expenditure which households are unable to meet with their own resources, requiring transfers from family members outside the household, and that being insured alone may not be as important as the type and extent of the health insurance benefits. These results are consistent with recent research from China, for example, that suggests social security benefits, and thus the affordability of care, vary considerably among disabled older adults in different socio-economic groups, with the poorest least likely to be able to afford long-term care (Lei et al., 2016). Transfers from adult children in China to their parents have also been found to be responsive to parents' demand for health services (and their income level) (Wu and Li, 2014). Our results may also be explained by the persistence of a culture of filial obligation in some countries included in this study (Aboderin, 2004, Aboderin, 2006). It is therefore surprising that the relationship between receiving net positive transfers and requiring long-term care was not statistically significant in India, where the obligation of sons to meet the long-term care needs of their parents has been provided as one reason for son preference in the country (Kadoya and Khan, 2016). One possible explanation for this is that transfers from adult children to ageing parents in India are common regardless of the long-term care needs, and therefore unaffected by the extent of these needs, or if perceived as an obligation, are not perceived as ‘transfers’. The relationship was also not statistically significant in Mexico, perhaps due to expanding coverage of the pension (Aguila et al., 2016), health insurance (Frenk et al., 2009), and poverty alleviation programmes (Valle, 2016) in that country.

There were other common factors influencing receipt of net positive transfers across the included countries. Receiving net positive transfers was significantly negatively associated with the proportion of the household members currently working, in every country except for Mexico. These results suggest that in these countries, social security systems are not sufficiently meeting the financial needs of those who are no longer in formal or informal employment. Our results also point to an important negative relationship between the proportion of the household that is male and odds of receiving net positive transfers in China and in Ghana. More research on access to public transfers from a gender perspective is needed in these countries, but our results are consistent with evidence from Nigeria suggesting that female-headed households are more likely to depend on others to finance health care costs, whereas male-headed households are more likely to have health care costs subsidised (Onah and Govender, 2014). Finally, mean household age was also associated with increased odds of receiving net positive transfers in China, Ghana, India and Mexico. It has been previously asserted that net family transfers increase with age in Asia and Latin America (Lee et al., 2010); our results suggest this may be the case in African countries as well.

While household need for long-term care appeared to influence the net flow of resources in the family, we did not find any relationship between the proportion of household needing care and the *amount* of transfer received, except for in Mexico. These results may suggest that there are limits to the ability of extended families to transfer resources. In other words, while the extended-family network may able to provide at least some economic support to cope with older family members’ long-term care needs, the amount of resources that can be mobilized is more likely to be driven by the networks’ own resources than by the care needs of the affected household. This interpretation is supported by our results in China, which suggest that the transfer amount is highest in the richest income quintile. However, we cannot assume that there is a correlation between the income of the recipient and source households. There is no statistically significant relationship between income quintile and amount of transfer received in the other countries. The positive effect in Mexico may be explained by the fact that, despite the expansion in social security provided to older Mexicans, when health-related expenditure does occur in this population, it tends to higher than the average population and largely driven by costly hospitalizations rather than ambulatory care (Gonzalez-Gonzalez et al., 2011).

Our findings should be interpreted in light of some limitations. First, our analysis relies on cross-sectional, self-reported financial data, which have been shown to be vulnerable to recall bias. Secondly, we cannot know for certain whether the family transfer received was used for long-term care expenditure, or for some other expense. Nevertheless, the strong significant positive relationship between needing long-term care and receiving positive transfers, after accounting for other covariates, suggests that a relationship between these two is likely. Future studies may consider collecting data that can identify which household expenditures are financed by family transfers. Finally, our analysis does not account for other means of support provided by adult children to ageing parents, such as co-residence and care-giving, which if accounted for would increase the observed amount of support provided. We also acknowledge that, while our paper focuses on family support received by older households, many older households also likely provide significant support to their adult children, for example through taking care of grandchildren, and for a comprehensive assessment of the economic impact of population ageing this support must be accounted for. Despite these limitations, our study presents strong evidence that, in selected LMICs, receiving family transfers is common among older households and is associated with requiring long-term care. Our results suggest that more comprehensive health care insurance and pensions for ageing populations are needed to protect extended families from the burden of costs associated with long-term care in LMICs. However, further research is needed within these countries, to better understand the drivers of the observed associations and to identify ways in which the financial protection of older adults’ long-term care needs can be improved.
